# Assessment of the degradation rates and effectiveness of different coated Mg-Zn-Ca alloy scaffolds for in vivo repair of critical-size bone defects

**DOI:** 10.1007/s10856-018-6145-2

**Published:** 2018-08-17

**Authors:** Nan Zhang, Dewei Zhao, Na Liu, Yunfeng Wu, Jiahui Yang, Yuefei Wang, Huanxin Xie, Ye Ji, Changlong Zhou, Jinpeng Zhuang, Yaming Wang, Jinglong Yan

**Affiliations:** 1 The Second Affiliated Hospital of Harbin Medical University, Harbin, Heilongjiang People’s Republic of China; 2 The Second Affiliated Hospital of Qiqihar Medical College, Qiqihar, Heilongjiang People’s Republic of China; 3 The Affiliated Zhongshan hospital of Dalian University, Dalian, Liaoning People’s Republic of China; 4 Harbin Institute of Technology, Harbin, Heilongjiang People’s Republic of China; 5 Qiqihar Medical College, Qiqihar, Heilongjiang People’s Republic of China

## Abstract

Surgical repair of bone defects remains challenging, and the search for alternative procedures is ongoing. Devices made of Mg for bone repair have received much attention owing to their good biocompatibility and mechanical properties. We developed a new type of scaffold made of a Mg-Zn-Ca alloy with a shape that mimics cortical bone and can be filled with morselized bone. We evaluated its durability and efficacy in a rabbit ulna-defect model. Three types of scaffold-surface coating were evaluated: group A, no coating; group B, a 10-μm microarc oxidation coating; group C, a hydrothermal duplex composite coating; and group D, an empty-defect control. X-ray and micro-computed tomography(micro-CT) images were acquired over 12 weeks to assess ulnar repair. A mechanical stress test indicated that bone repair within each group improved significantly over time (P < 0.01). The degradation behavior of the different scaffolds was assessed by micro-CT and quantified according to the amount of hydrogen gas generated; these measurements indicated that the group C scaffold better resisted corrosion than did the other scaffold types (P < 0.05). Calcein fluorescence and histology revealed that greater mineral densities and better bone responses were achieved for groups B and C than for group A, with group C providing the best response. In conclusion, our Mg-Zn-Ca-alloy scaffold effectively aided bone repair. The group C scaffold exhibited the best corrosion resistance and osteogenesis properties, making it a candidate scaffold for repair of bone defects.

## Introduction

Presently, treatment of critically sized and large defects in long bones of humans caused by trauma, infection, or a tumor remains a challenging undertaking for orthopedic surgeons [1, 2]. Available repair modalities include autogeneic, allogeneic [3, 4], and vascularized bone grafts [5]. Although these treatments have achieved positive outcomes, they are not without drawbacks, indicating the need for improvement] [4, 6, 7]. To effectively repair defects in long bones, Cobos et al. [8] described a simple and effective technique that uses a titanium mesh cage in combination with a bone graft to repair critical-size bone defects [9–11]; however, it is not possible to remove the inert titanium mesh after it becomes covered with bone, and the continual presence of titanium mesh in a bone may cause stress shielding and secondary bone absorption [12]. Thus, how to pair new materials with known surgical grafting options remains a major clinical problem.

Magnesium and its alloys have been extensively investigated as possible materials for bone implants because of their excellent mechanical properties [13–15]. The most intriguing advantage of a Mg alloy is that it can be tuned to resorb after completion of the bone-healing process, which would eliminate the need for a second surgery to remove it, thereby reducing the risk of infection, discomfort, and cost. However, the use of a Mg-alloy scaffold to repair a large segmental bone defect has not been reported. Therefore, we fabricated a scaffold made of a Mg-Zn-Ca alloy that is hollow and cylindrical, a shape that mimics a cortical bone and allows filling of the internal space with finely ground autologous morselized bone. We hypothesized that the scaffold could be used as a substitute for the titanium mesh–type scaffold currently used to repair bone defects. This scaffold would effectively reduce or obviate postoperative complications inherent in the use of the titanium mesh cage, which includes stress shielding and removal complications, and might promote bone regeneration related to the presence of Mg^2+^, which can stimulate more osteoblastic response and enhance porosis [16], so that a bone defect could be repaired. The purposes of the present study were, therefore, as follows. To evaluate the durability of different scaffold compositions in vivo and to assess the relative efficacies of the scaffolds for repairing long-bone defects.

## Materials and methods

### Design of different Mg-alloy scaffolds and the coatings

**Fig. 1:**
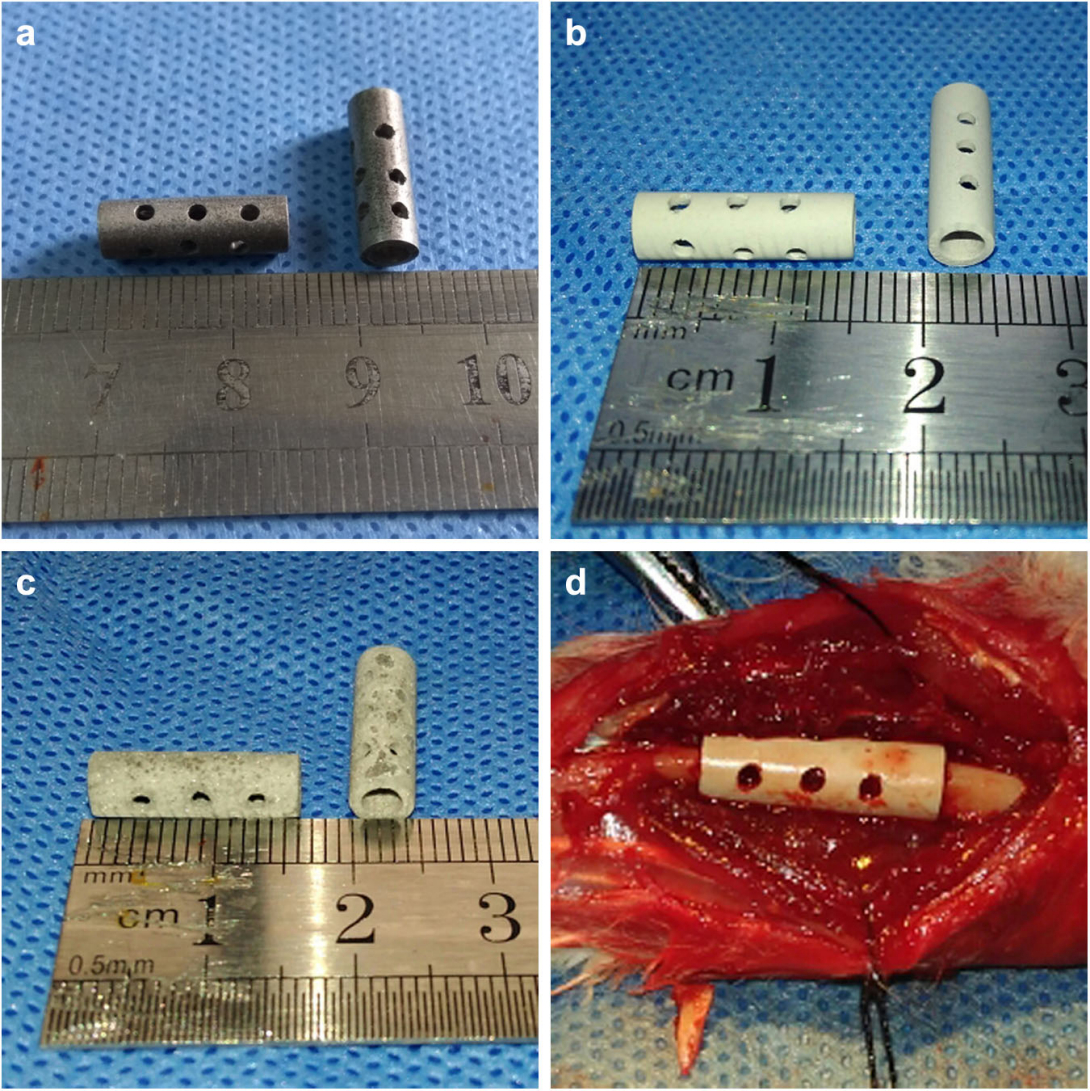
The uncoated and coated Mg-alloy scaffolds used in this study. The uncoated, 10-μm MAO-coated, and hydrothermal duplex-coated scaffolds are shown in **a**, **b**, and **c**, respectively. **d** Positioning of a scaffold containing autogenous morselized bone in a broken rabbit ulna

The basic Mg-alloy scaffold contained 2.5–3.0% (w/w) Zn, 0.5–1.5% (w/w) Ca, and 0.5% (w/w) of a mixture of rare elements in pure Mg and was manufactured using a die-cast technique as a cylinder of length 15 mm, outer diameter 5 mm, and inner diameter 3 mm, with 12 holes (each 1-mm diameter) drilled into the outside wall (Fig. 1a). The 10-μm microarc oxidation (MAO) bio-ceramic coat (Fig. 1b) and a hydrothermal duplex coat were synthesized (Fig. 1c) as described previously [17]. Each scaffold underwent ethylene-oxide gas sterilization before implantation.

### Surgical procedure

All animal experiments were approved by the Harbin Medical University Institutional Animal Care and Use Committee. Adult New Zealand White rabbits (36 male, 36 female; mean age, 6.23 ± 0.37 months; mean weight 2.56 ± 0.25 kg) were randomly assigned to one of four groups (18 per group), with group A receiving the uncoated Mg-alloy scaffold, group B receiving the 10-μm MAO-coated scaffold, group C receiving the hydrothermal duplex layer scaffold, and group D (control) not receiving a scaffold. The rabbits were anesthetized intravenously through their ear veins with 3% (w/v) pentobarbital sodium (30 mg/kg). Next, both forearms of the rabbits were shaved and disinfected, and their ulnae exposed. A metal caliper was placed over each ulna to mark the break positions, and two ulna osteotomies was performed ~15 mm apart. During this procedure, care was taken not to touch the radius. The bone fragments generated during the osteotomies were removed and minced into small particles (diameter, 0.5–1 mm). Each scaffold was filled with compacted, morselized bone and was then carefully placed into the ulna at the position where the bone had been removed (Fig. 1d). The bone in the control group was not filled with any material. The reason for using this defect model is that internal fixation of the ulna is not needed because the adjacent radius is strong enough to support the animal when it moves [18]. Because each adjacent radius was intact, and the scaffold was embedded in one broken end of the ulna, the implant was stable. In addition, the soft tissue and muscle adjacent to each scaffold were carefully sutured to help stabilize the scaffold. Immediately following surgery, the animals were allowed to resume their normal movement and behavior, including bearing their weight on their forearms.

The rabbits were housed for 4–12 weeks in stainless steel cages (one rabbit per cage) at 22 °C, 60% humidity, and under a 12-h light/dark cycle in a ventilated room. Water and food were available ad libitum. On each of the 3 days following the procedure, the rabbits were given a subcutaneous injection of 10 mg/kg penicillin. Each rabbit’s stitches were removed 10 days after the surgery. At 4, 8, and 12 weeks after surgery, six rabbits per group were anesthetized and sacrificed by exsanguination. Their bilateral forearms (12 per group per time point) were collected and frozen at −20 °C for further testing.

### X-ray imaging

Scaffold placement and the forearm bone-healing process were imaged using an X-ray scanner (Faxitron, USA; 110 kV; anode current, 500 μA) at 4, 8, and 12 week after surgery. The lateral X-ray images of the forearm healing process were assessed and scored by three experienced, independent investigators who used the criteria reported in [19]. Due to the radius exit, which can induce new bone regeneration in the medial position of the bone defect, This can lead to a false-positive scoring in X-ray image,Therefore, we only evaluated the X-ray images of the cortical bone opposite the radial side. Scores ranged from 0 (no healing) to 12 (restoration of normal bone), a score of 7 was considered the defect was preliminary reconstruction.

### Micro-computed tomography (micro-CT)

Each rabbit’s forearms post-implant were scanned using an Inveon Micro-CT system (Siemens Medical Solutions, Germany). Scans were performed with an X-ray voltage of 80 kV, an anode current of 500 μA, and a 1000-ms exposure time. Three-dimensional (3D) images were generated from the acquired 2D lateral projections using Inveon software. In each image, the scaffold could be distinguished from the surrounding soft and hard tissues based on differences in absorption coefficients (which for bone was the equivalent of mineral density). The residual scaffold volumes were each determined by 3D morphometric analysis, and the corresponding corrosion rate was calculated using the micro-CT–evaluated Mg-volume loss as follows:

$${\mathrm{CR = }}\Delta V{\mathrm{/}}\left( {{\mathrm{At}}} \right),$$

where CR is the corrosion rate in mm/y, Δ*V* is the absolute value of the decrease in the volume of the Mg-alloy scaffold in mm^3^ at the given time, A is the surface area in mm^2^, and t is time in years.

### Subcutaneous production of hydrogen gas

It is well known that hydrogen gas was produced during the degradation of magnesium alloys in the body [14–16]. Depending on the local environment of an implant and available blood flow, hydrogen gas may or may not accumulate at the implant site; notably, rapid corrosion and/or insufficient gas removal will lead to gas accumulation [20]. Thus, when each rabbit was anesthetized as described above, any subcutaneous gas bubbles present were punctured using a LOGIQ 7 color-Doppler ultrasound diagnostic system (GE, USA) and collected in a syringe to indirectly quantify the extent to which the scaffold had degraded.

### Mechanical strength test of newly formed bone

A three-point bend test was used to evaluate the relative strength of newly formed bone. Forearms that had been removed and frozen were defrosted and carefully dissected to remove the soft tissue that overlaid the bone. To exclude contributions of the radius-ulna fusion region to the test, each radius received two 1-mm in-depth transverse cuts 5 mm beyond the ends of the original defect region with an oscillating saw [21]. The test was performed using an AG-X plus 20-kN platform (Shimadzu, Japan) beginning with a 50-N load and then increasing the load at a rate of 3 mm/min. The fracture stress of each ulna was calculated as:

$$\sigma _{{\mathrm{bb}}} = 8\,{\mathrm{FL/\pi d}}^3,$$

where σ_bb_ is the fracture stress in N/mm^2^, F is the fracture load in N, L is the span of the three-point bend test, which was 30 mm, and d is the diameter of the ulna (mm).

### Calcein fluorescence

To characterize mineralization of newly formed bone, 3 or 4 days prior to sacrifice, a solution of 1% (w/v) calcein in 2% (w/v) NaHCO_3_ (10 mg/kg, Sigma-Aldrich, USA) was injected under the skin. Then, the harvested ulnae were fixed in 75% (v/v) ethanol. samples were embedded and polymerized in methyl methacrylate resin (Technovit 7200, Kulzer, Germany). The resin blocks were cut using a EXAKT/E300CP microtome (Leica, Germany) with a tungsten carbide blade into uncalcified sections of ~120 μm. Next, the sections were polished to a thickness of ~50 μm using an cutting-grinding system (EXAKT/E400CS, Leica). Details of the bone sections were visualized through a DM4000B fluorescence microscope (Leica), and the extent of mineral deposition evaluated according to their fluorescence intensities.

### Histology

Bone samples were histologically processed to micromorphologically observe newly formed bone tissue surrounded the scaffolds. Samples were sectioned as described in Section 2.7, decalcified, and stained with Van Gieson’s picrofuchsin solution (Sigma-Aldrich) to visualize osteocytes, chondrocytes, and surrounding soft tissue. The sections were visualized and photographed through an optical microscope (Model X71, Olympus, Japan).

### Statistical analysis

Statistical analysis was performed using Prism 6.0 software (GraphPad, USA). All results are reported as the mean ± the standard deviation. All groups were compared using one-way ANOVA followed by Tukey’s test. The X-ray scores, the corrosion rates, and mechanical stress test results were compared at 4, 8, and 12 weeks (12 forearms per time point). In addition, the volumes of the gas bubbles collected at weeks 2, 4, 8, and 12 were compared (12 forearms per time). Statistical significance was defined as P < 0.05 or as P < 0.01.

## Results

### Evaluation of X-ray images

**Fig. 2:**
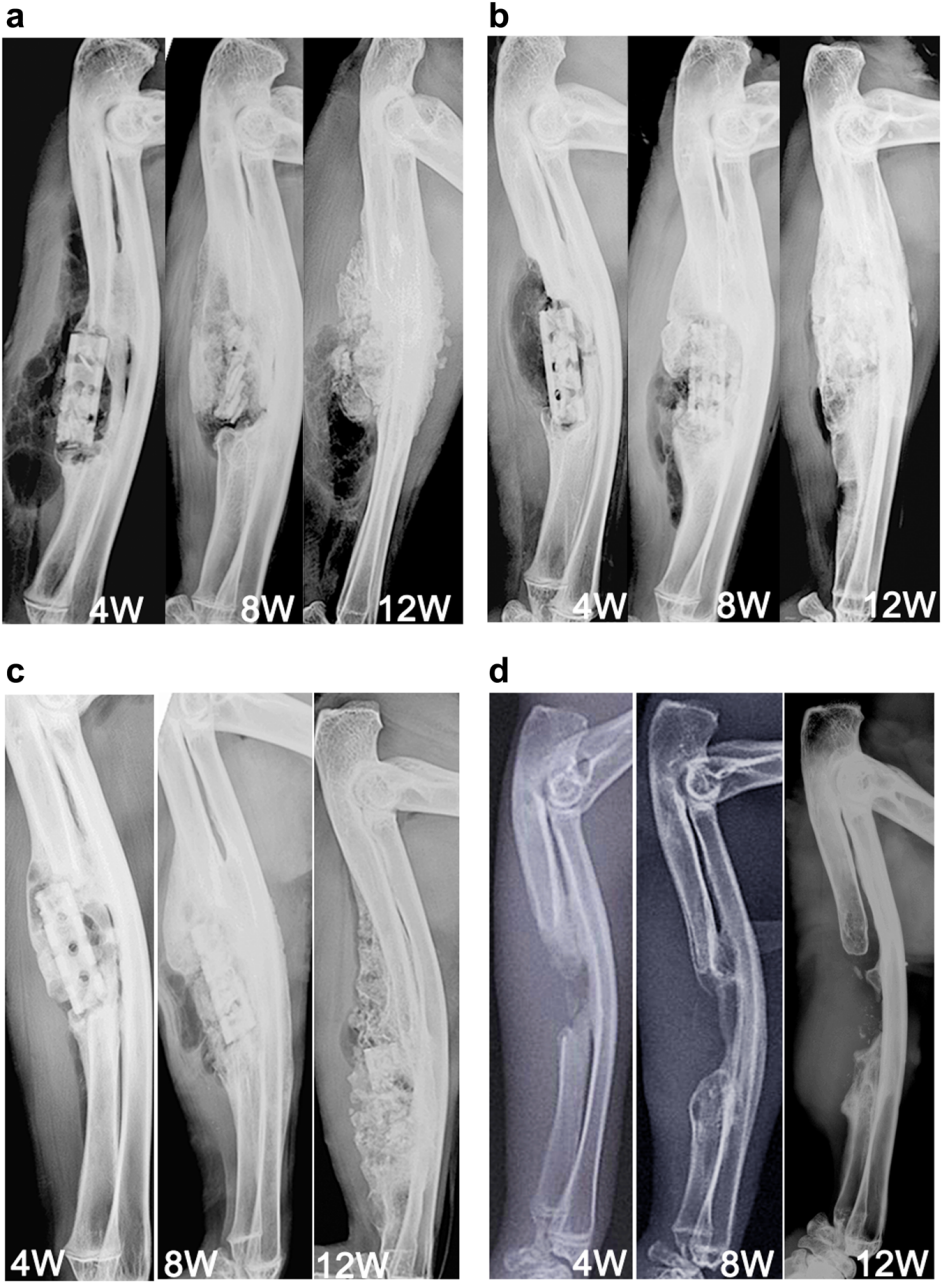
X-ray images of scaffolds in rabbit ulnae at 4, 8, and 12 weeks after implantation. Implanted uncoated, 10-μm MAO-coated, and hydrothermal duplex-coated scaffolds are shown in **a**, **b**, and **c**, respectively. **d** X-ray images showing the empty voids in the ulnae of the control group

**Fig. 3:**
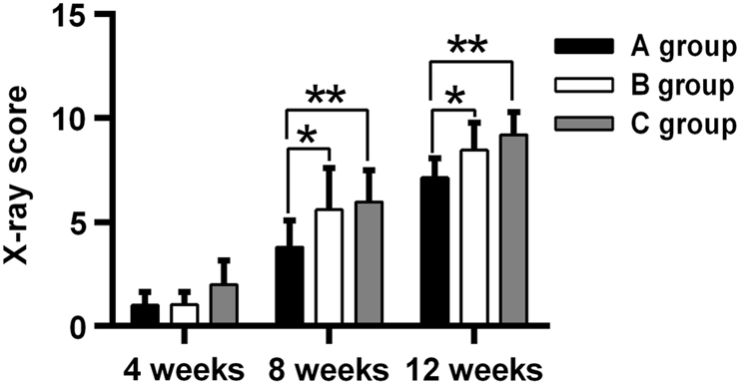
X-ray images for the different coated scaffolds at weeks 4, 8, and 12 weeks after surgery. Group A, un**c**oated scaffold. Group B, 10-μm MAO-coated scaffold. Group C, hydrothermal duplex-coated scaffold. *P < 0.05; **P < 0.01

Figure 2 shows X-ray images of bone that reformed around the scaffolds and in the empty void of the control group 4, 8, and 12 weeks after surgery. The formation of new bone and bone remodeling at the implantation sites can be seen in all groups at week 4. The three types of scaffolds apparently were still mostly intact in the forearms at week 4. By week 8, even more new bone was apparent within the defects and at the bone-defect junctions. The uncoated scaffolds had begun to degrade at that time. At week 12, abundant and mature bone tissue had formed in the regions of the defects; indeed bony bridges were apparent between the scaffolds and the defects, and the fracture lines had disappeared when the integrity and continuity of the ulnae were restored. However, both types of coated scaffolds (Figs. 2b, c) showed better bone generation around the defect area at week 12 than the uncoated scaffold (Fig. 2a). Conversely, the ulnae of the control group had not been repaired by week 12 (Fig. 2d). As is well known, The X-ray scores for the three groups are presented in Table 1. No significant difference was apparent among the groups at week 4 (Fig. 3); at weeks 8 and 12, however, the scores for groups B and C were significantly greater than for group A (P < 0.01 OR 0.05 for B vs. A, and P < 0.01 for C vs. A). In addition, the scores for group C tended to be greater than for group B, although the scores for the two groups were not significantly different.

**Table 1:** X-ray image scores for different coated scaffold at week 4, 8, and 12 weeks after surgery

	Week 4	Week 8	Week 12
Uncoated scaffold	1.01 ± 0.64	3.81 ± 1.28	7.14 ± 1.02
MAO coated scaffold	1.04 ± 0.6	5.64 ± 1.89	8.33 ± 1.35
Hydrothermal coated scaffold	1.65 ± 0.96	6.28 ± 1.56	9.4 ± 1.13

### Micro-CT images

**Fig. 4:**
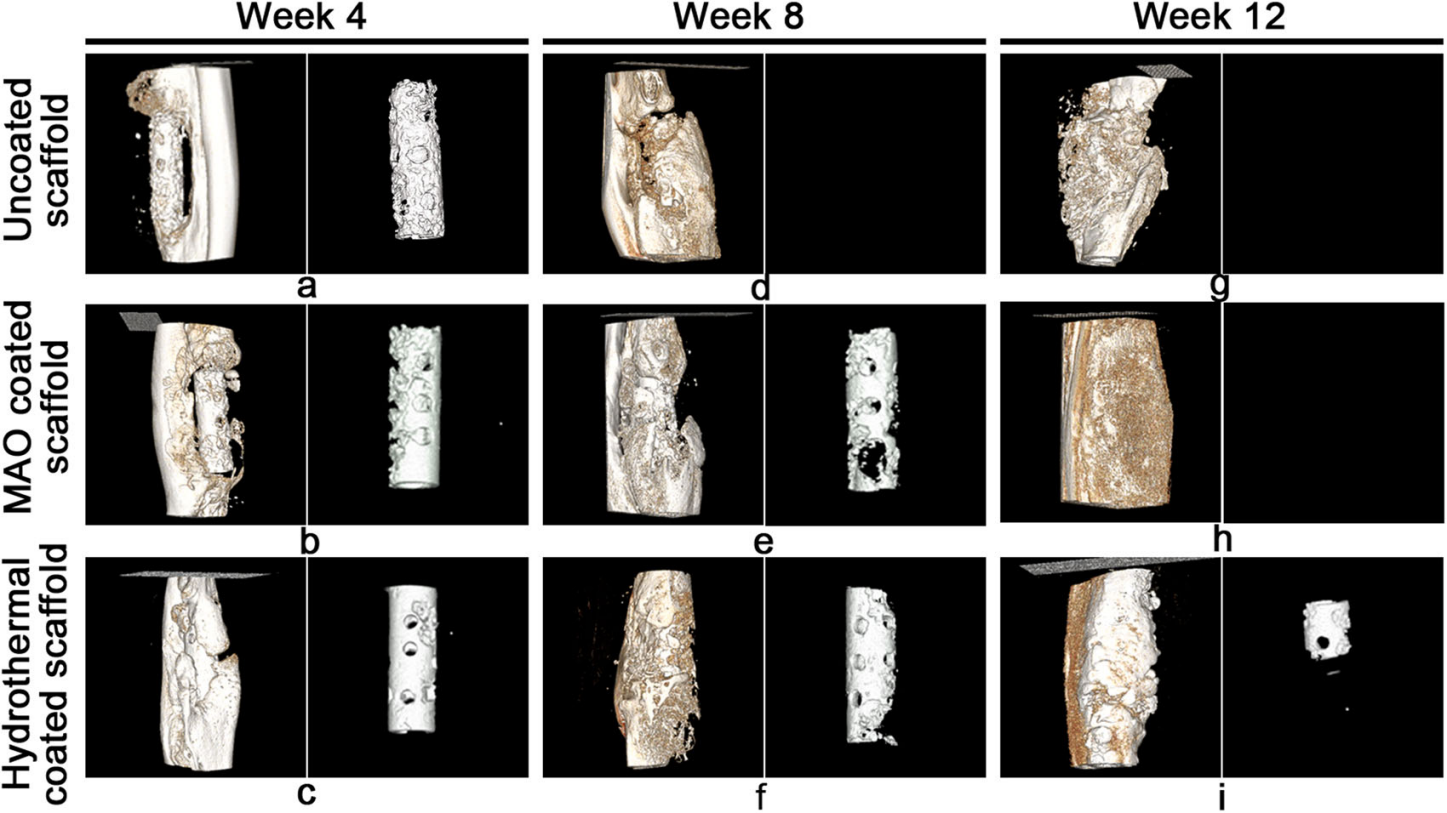
Micro-CT images showing new bone formation and the degree of degradation of the three types of Mg-alloy scaffolds at weeks 4, 8, and 12 after surgery

The corrosion rate (CR) and the volume-loss percentage for each ulna containing a scaffold were calculated using its 3D micro-CT images (Fig. 4 and Table 2). By week 4, the mean CR value for the uncoated scaffolds was 2.58 ± 0.34 mm/y, and this value was the greatest among the three groups. Significant differences for the CR values were found among the three groups (P < 0.01). At week 8, the mean CR value for the uncoated scaffolds was 2.52 ± 0.08 mm/y and the mean volume-loss percentage was 98.8 ± 3.22%, implying that the scaffolds had completely corroded (Fig. 4d). The mean CR value for group C had increased from 0.71 ± 0.2 mm/y at 4 week to 1.03 ± 0.11 mm/y by 8 week. Significant differences were found among the three experimental groups (P < 0.01). By week 12, additional bone formation was found within and around the scaffolds, throughout the defects, and at the bone-defect junctions (Fig. 4). The scaffolds and defects were completely covered with new bone. Notably, more spongy callus formation in the defect area was seen in the uncoated (Fig. 4g) and MAO-coated (Fig. 4h) scaffolds than those covered with the hydrothermal duplex coat (Fig. 4i). By week 12, the volume loss for the scaffolds of group C was 74.06 ± 5.86%, and its mean CR value was 1.26 ± 0.1 mm/y, which was significantly smaller than that for group B (P < 0.05). In accordance with the smaller CR value for group C than for group B, the scaffolds for group B were no longer observable, whereas the scaffolds of group C had not completely degraded. Interestingly, the mean CR value for the scaffolds of group C gradually and significantly increased with time (P < 0.01).

**Table 2:** Percentage volume loss (DV) and corrosion rate (CR) for the uncoated and coated scaffolds at weeks 4, 8, and 12 after surgery

	Week 4	Week 8	Week 12
	ΔV (%) CR(mm/y)	ΔV (%) CR(mm/y)	ΔV (%) CR(mm/y)
Group A	50.36 ± 6.75% 2.58 ± 0.34	98.8 ± 3.22% 2.52 ± 0.08	— —
Group B	33.72 ± 6.08% 1.75 ± 0.31	71.92 ± 4.84% 1.84 ± 0.12	98.6 ± 2.7% 1.68 ± 0.05
Group C	13.9 ± 3.87% 0.71 ± 0.2	40.15 ± 4.14% 1.03 ± 0.11	74.06 ± 5.86% 1.26 ± 0.1

Group A, uncoated scaffold. Group B, 10-μm MAO coated scaffold. Group C, hydrothermal coated scaffold

### Hydrogen gas formation as a measure of scaffold corrosion

**Fig. 5:**
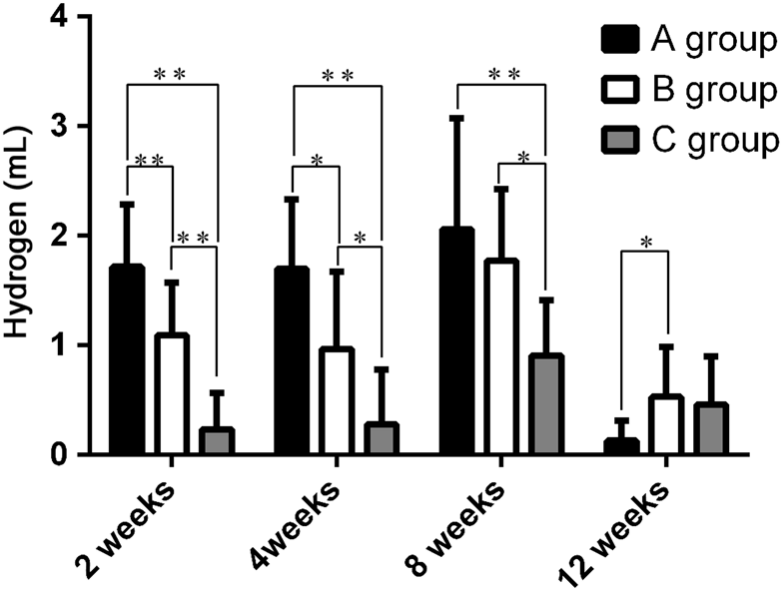
Volumes of hydrogen gas extracted from the volumes surrounding each of the three types of Mg-alloy scaffolds at weeks 2, 4, 8, and 12 after surgery. *P < 0.05; **P < 0.01. Group A, uncoated scaffold. Group B, 10-μm MAO-coated scaffold. Group C, hydrothermal duplex-coated scaffold

Gas bubbles that did not contain blood or other bodily fluids were easily removed with a sterile syringe, and the bubbles, being sterile, had not caused infection. Gas volumes were measured at weeks 2, 4, 8, and 12 (Fig. 5), with the results indicating significant differences between all pairs of groups at weeks 2 and 4 (P < 0.05). The gas volume was greater for group A at weeks 2 and 4 than for the other two groups, and the gas volume was the least for group C at the same time. By week 8, the volumes of gas for groups A and B had increased dramatically, although the difference between the two groups was not significant. Conversely, the gas generated by the group C scaffolds was significantly less than that for groups A and B (P < 0.05). At week 12, the gas volumes from groups A and B were significantly less than previously measured, and the gas from group C had slightly increased, although only the volumes of groups A and B differed significantly (P < 0.05).

### Biomechanical testing

**Fig. 6:**
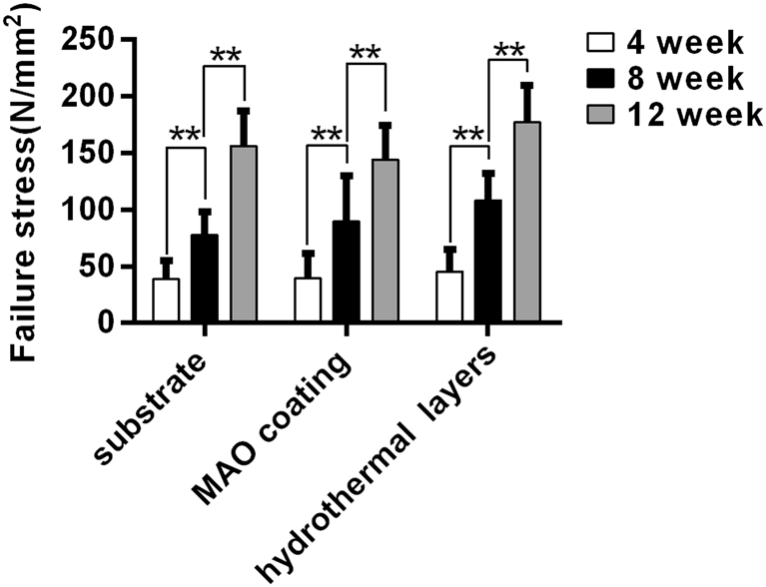
Mechanical stress test results for the uncoated and coated scaffolds at weeks 4, 8, and 12 after surgery. *P < 0.05; **P < 0.01

The biomechanical testing were used to evaluate the healing conditions of different scaffold groups at different stages. The mechanical failure-stress results are shown in Fig. 6. Significant differences within each group were found at weeks 4, 8, and 12 (P < 0.01). For each time point, no significant differences were found when the values were compared across the groups. This may be due to the fact that the healing rate of three groups is relatively close, although different, but it is not obvious in biomechanics. For instance, at 12 week, the ulna defect had been reconstructed effectively in three groups. so there was no significant difference to be found between groups. In addition, the results of mechanical experiments are affected by many factors [22], such as individual difference, ambient conditions, applied/indentation load, and geometry and dimension of the specimen, these may induce the failure of mechanical testing to accurately reflect the slight differences in reality. However, Significant differences within each group were found at weeks 4, 8, and 12 (P < 0.01). This result suggests that the mechanical property of unla in all groups had been recovered signally in the healing process, and demonstrated that this new Mg-Zn-Ca scaffold can repair effectively critical-size bone defect.

### Mineralization

**Fig. 7:**
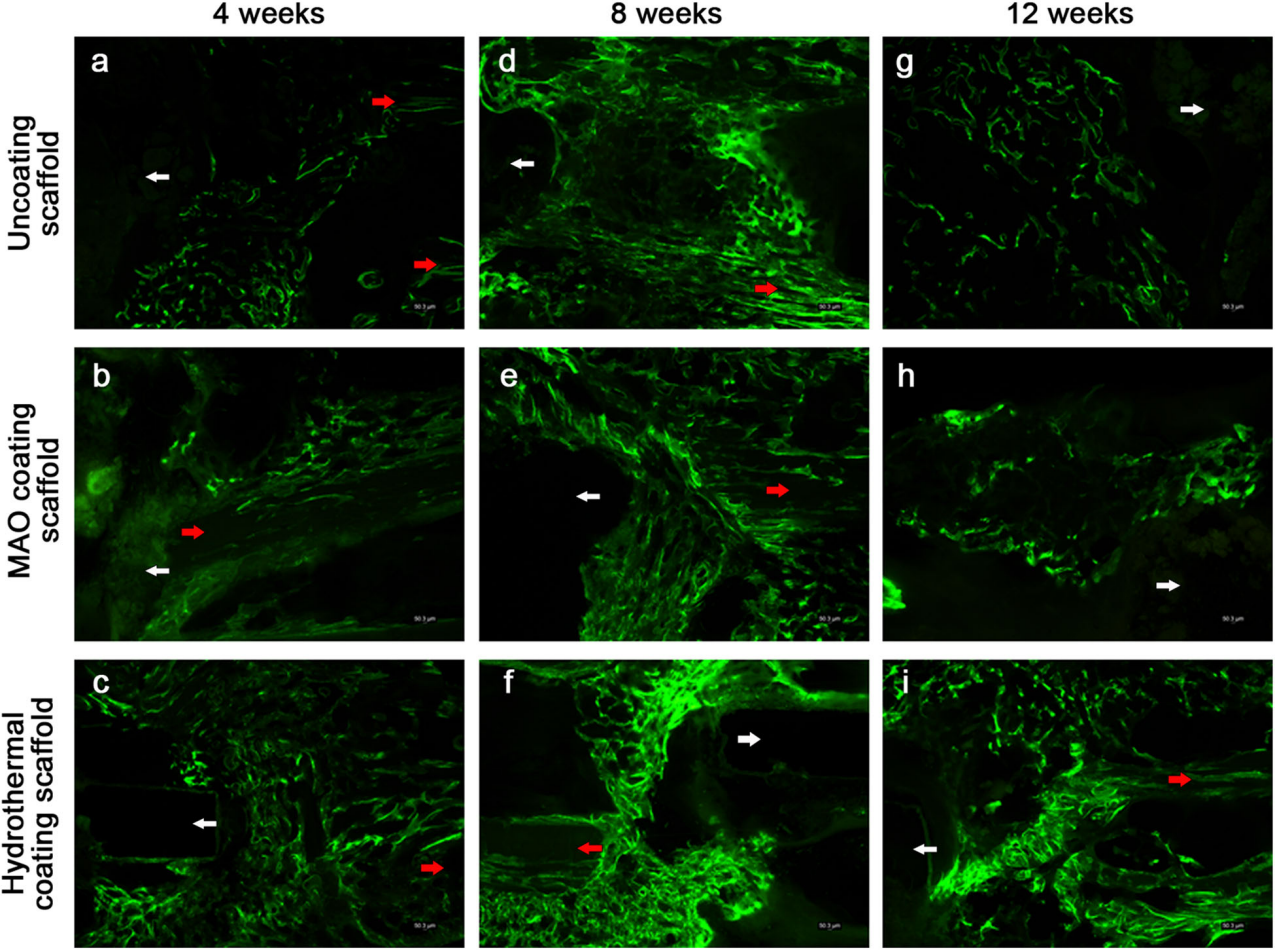
Images of calcein fluorescence (green) around the scaffold-bone interfaces at weeks 4, 8, and 12 after surgery. The fluorescence identifies sedimentary [Ca^2+^ OR calcium] and newly formed bone. White arrows indicate the original positions of the scaffolds. Red arrows indicate the cut ends of the ulnae

The fluoroscopic calcein images of the bone tissue around the scaffolds at weeks 4, 8, and 12 are shown in Fig. 7. The bright green calcein fluorescence is indicative of the presence of sedimentary calcium and newly formed bone [16]. By week 4, the fluorescence intensity around the scaffolds was similar for groups A, B, and C. At week 8, the fluorescence intensity of every group was greater than it had been at week 4. Interestingly, at week 12, the fluorescence around the scaffolds of group A and B was more intense than for the same two groups at 8 week. The mean intensity was greater for group C than for groups A and B, and for group C the intensity was similar at weeks 8 and 12.

### Histology

**Fig. 8:**
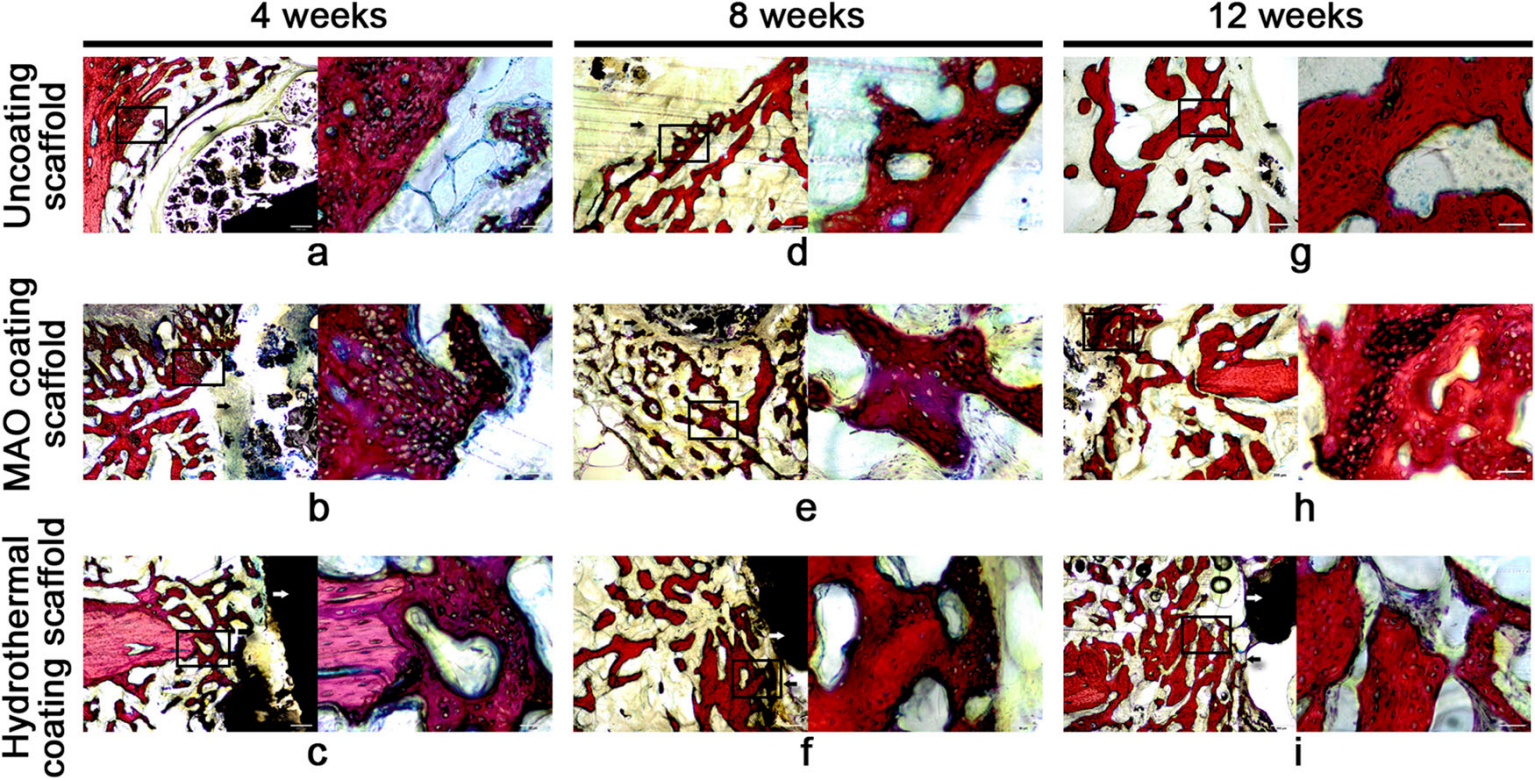
Histological images of the scaffold/bone interfaces at weeks 4, 8, and 12 after surgery. White arrows indicate the scaffold positions. Black arrows identify fibroblast bands. In each panel, an enlargement of the area enclosed in the black square in the left image is shown on the right to document new bone tissue. Cells colored blue are chondrocytes, and cells colored red are osteocytes

Figure 8 shows histological images of the tissues at the edges of the scaffolds at weeks 4, 8, and 12. Many chondrocytes and new, small-island bone structures were observed for the three groups with the new osteocytes being immature and arranged in an irregular manner at week 4. At the same time, large continuous fibroblast bands were seen around the uncoated scaffolds (Fig. 7a). Again, at week 4, many chondrocytes and relatively small fibroblasts were apparent with a relatively large amount of newly formed bone in group B (Fig. 7b), and the number of fibroblast bands was smallest for group C (Fig. 7c). By week 8, in comparison with week 4, a greater number of mature bone cells were observed for all groups, although the number of chondrocytes had decreased. The fibroblast bands around the group C scaffolds (Fig. 7f) were thinner than those of groups A (Fig. 7d) and B (Fig. 7e). At week 12, all newly formed bone cells had matured and were arranged symmetrically around the scaffolds of groups A and B (Fig. 7g, h, respectively), and the numbers of chondrocytes surrounding the scaffolds had decreased further in both groups. In line with the aforementioned observations, the fibroblasts bands around the scaffolds of groups A and B were thinner than seen at week 8. Conversely, although correctly aligned mature bone cells and osteoid tissue contacts around the scaffolds of group C were seen, cartilage cells and immature bone cells were also found (Fig. 7i), suggesting that bone remodeling around the group C scaffolds was still active at week 12.

## Discussion

### Degradation of scaffolds

Because the Mg-Zn-Ca alloy has excellent mechanical and biocompatibility properties, it may serve as a material for bone implants in the future [23–26]. However, a drawback to its use is that its corrosion rate cannot be effectively controlled once placed in a body. For this report, we showed that, relative to the routine healing period for a bone fracture, our uncoated scaffold degraded much more rapidly and had completely degraded by 8 weeks post implantation. The probable reason for the rapid degradation is the presence of body fluids and soft tissue found in the region of the bone defect. Previous studies also demonstrated that corrosion of Mg alloys depends on their liquid environments [19]. As an initial attempt to slow the degradation of an implanted, uncoated scaffold, we coated scaffolds with a 10-μm MAO bio-ceramic layer. The MAO layer contains MgSiO_3_, Mg_2_SiO_4_, CaSiO_3_, and MgO, which are expected to improve the resistance of the scaffold to corrosion and its biocompatibility [27–29]. The CR values of the MAO-coated scaffolds were significantly smaller than those of uncoated scaffolds, and significantly less hydrogen was collected from the implanted MAO-coated scaffold sites compared with the implanted uncoated scaffold sites at weeks 2 and 4, which demonstrated that the MAO coat effectively reduced degradation of the implanted device. Interestingly, no significant difference was observed for hydrogen accumulation at the MAO-coated and uncoated scaffolds at week 8, suggesting that the protective effect of the MAO layer no longer existed by then. This decrease of protection with time may be ascribed to the presence of micropores and crack defects in the MAO layer, which initially allow body-fluid ions to attack the scaffold but with corrosion products subsequently blocking further corrosion [30, 31]. Consequently, degradation of the MAO-coated scaffold accelerated with time such that a mean volume loss of 98.6 ± 2.7% was found at week 12, indicating that the corrosion resistance of the MAO coat needed improvement.

**Fig. 9:**
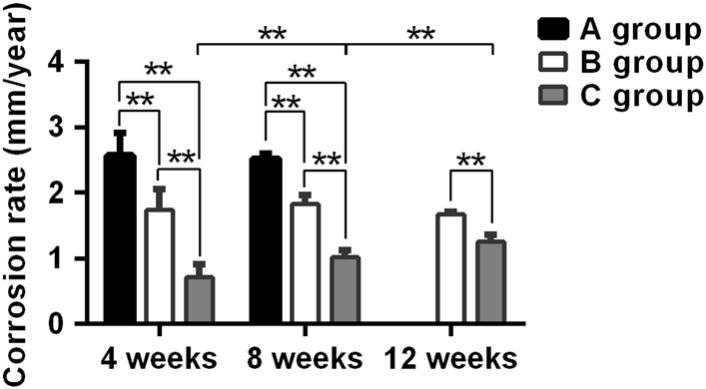
Corrosion rates of the three types of Mg alloy scaffolds at weeks 4, 8, and 12 post-implantation. *P < 0.05; **P < 0.01. Group A, uncoated scaffold. Group B, 10-μm MAO-coated scaffold. Group C, hydrothermal duplex-coated scaffold

To seal any micropores and cracks that open up to the MAO surface, which would improve the resistance of the scaffold to corrosion, a bioactive 60-μm thick hydrothermal duplex layer was placed over the MAO layer. We had previously demonstrated that the hydrothermal composite layer improved the corrosion resistance of the Mg-alloy scaffold in an in vitro test [17]. In this in vivo study, we found that the hydrothermal duplex-coated scaffold volume loss was 74.06 ± 5.86% by 12 weeks of implantation. Consequently, the X-ray and micro-CT images of the forearms containing the hydrothermal duplex-coated scaffolds indicated that partially degraded scaffolds were present at week 12. We also found that the CRs of the hydrothermal duplex-coated scaffolds were significantly less than those of the MAO-coated scaffolds throughout the experiment, which demonstrated that the hydrothermal duplex coating effectively delayed degradation of an implanted scaffold. However, we also found that the mean CR value for the hydrothermal duplex-coated scaffold increased significantly with time (Fig. 9; P < 0.05). Nonetheless, the amounts of hydrogen gas generated by the hydrothermal duplex-coated scaffolds were relatively small and were constant with time; during the experiment; further, only a small amount of gas existed at the interface between soft tissue surfaces and the hydrothermal duplex-coated scaffolds, suggesting that the gradually increasing corrosion rate found for the scaffolds may have not been caused by an increase in hydrogen production as the produced hydrogen may have been absorbed by the body. In summary, our results indicate that addition of the hydrothermal layer over that of the MAO-coated scaffold may significantly improve its corrosion resistance in vivo.

### Osteogenesis promoted by the coated scaffolds

Previously, a Mg alloy was demonstrated to have good osteoconduction and osteoinduction properties [32, 33]. When released in the body, Mg^2+^ may promote osteoblast proliferation and differentiation [34, 35], may enhance Ca^2+^ mineralization surrounding the implant [16], and may accelerate osteoanagenesis and osseointegration [36]. Our current study reveals that, as the scaffolds of the three groups degraded, new bone formation and remodeling occurred. By week 12, the integrity and repair of the ulnae were complete in the three groups, and these findings were supported by the three-point bending test results and suggested that the scaffold with or without a coat probably could effectively repair a large bone defect and recover the mechanical strength of a repaired ulna. Our results are in agreement with those of Smith and colleagues [37] who used an AZ31-alloy scaffold to repair bone defects in rabbits and who found that, at 12 weeks post-implantation, the defects were essentially repaired. Even though our mechanical test revealed no significant differences among the three types of scaffolds, the hydrothermal duplex-coated scaffolds seemed to retain a more intact structural appearance than did the uncoated and MAO-coated scaffolds according to the X-ray and micro-CT images. Improved remodeling and less spongy callus formation of new bone were observed around the hydrothermal duplex-coated scaffolds, which indicated that the new bone-growth response to the hydrothermal duplex-coated scaffolds was better than that of the MAO-coated scaffold.

The new bone-growth responses were perhaps better for the hydrothermal duplex-coated scaffolds than for the uncoated and MAO-coated scaffold for the following reasons. Because the bio-mimetic, hydrothermal-duplex coat more effectively inhibited corrosion of the underlying scaffold, new bone growth around and within the scaffold (generated from the morselized bone) was more likely to occur. In addition, the relatively intact scaffold was more able to resist mechanical destabilization axially and linearly during the healing process. Stabilization would also have promoted vascularization and morselized graft-bone integration. Moreover, although the coatings decreased the degradation rate of the Mg alloy, the steady release of Mg^2+^ into the surrounding soft tissues may have induced substantial mineralization. In our study, the calcein fluorescence intensity was similar in the images of bones from the three groups at week 4, which indicated that the amount of sedimented Ca^2+^ OR Ca^2+^ sediment present and newly formed bone tissues in all three groups were similar at that time. We found that the mineral densities around the uncoated and MAO-coated scaffolds were greatest at week 8 and had decreased by week 12, whereas for the hydrothermal duplex-coated scaffolds, the fluorescence intensities were similar at weeks 8 and 12, suggesting that the hydrothermal coating continued to stimulate mineralization at least until week 12. More importantly, the hydrothermal duplex coat enhanced the biocompatibility of the Mg-alloy scaffold and reduced the potential for foreign-body rejection more so than did the MAO coat. Osteoblasts and chondroblasts have been found to more easily proliferate and adhere to the bio-mimetic-type surface [38, 39]. Our histological study revealed that the number of mature osteocytes had increased, and the number of chondrocytes had decreased around the uncoated and MAO-coated scaffolds by week 8 and that mature osteocytes had arranged themselves in a regular manner around the scaffolds. Conversely, more immature bone cells and chondrocytes were present surrounding the hydrothermal duplex-coated scaffolds at that time. By week 12, more mature and regularly aligned osteocytes were found in the vicinity of the three types of scaffolds than before, although few chondrocytes were observed surrounding the uncoated and MAO-coated scaffolds. However, a small number of immature bone and cartilage cells were seen around the hydrothermal duplex-coated scaffolds, suggesting that bone formation was still occurring at week 12. Conversely, the fibroblast bands that were present near the hydrothermal duplex-coated scaffolds were thinner than those of the other two groups throughout the experiment. The presence of a relatively larger number of osteoblasts may reduce the number of fibroblast bands whose appearance is a normal reaction to foreign bodies [40]. Fibroblast bands near a scaffold may attract lymphocytes and allow for infiltration of inflammatory factors, thereby restricting osteoblast proliferation and differentiation and consequently inhibiting new bone formation surrounding the scaffold [35]. Our study suggests that the hydrothermal duplex coat could effectively stimulate osteoblast adhesion and reduce foreign-body reactions, thereby accelerating healing. Finally, hydrogen gas generation may have been confined to local bone formation, and although most of the hydrogen bubbles were removed upon puncturing, residual hydrogen was observed in the X-ray images. Because the uncoated and MAO-coated scaffolds were degraded more rapidly than the hydrothermal duplex-coated scaffolds, more hydrogen was released from the first two types of scaffold, and it could then accumulate between the newly formed bone and the surrounding soft tissue. The residual gas may have increased the pH value of the local environment, thereby affecting the activity of the osteoblasts [33] and consequently the quality of new bone. Although we did not find a negative effect on the osteoblast quality according to the mechanical stress test, the micro-CT images revealed that a large amount of hydrogen gas accumulated in the space that might have been filled with newly formed bone and that more spongy callus formed in the defect areas of the uncoated and MAO-coated scaffolds. This suggests that the presence of gas may have affected the quality of the newly formed bone. In summary, our study demonstrates that the hydrothermal duplex coat may improve the osteogenesis effect of the Mg-alloy scaffold.

Our study has certain shortcomings. First, we only monitored repair of the bone defect over a relatively short period. Consequently, we cannot comment on the long-term stability of the repair and further experimentation will be needed to address this issue. Second, micro-CT imaging was not used to determine the ratio of bone volume to total volume(BV/TV) in the defect, which may be used as an additional measure of new bone formation. In addition, because the hydrothermal duplex-coated scaffold produced some hydrogen gas that accumulated in the body, it cannot be used clinically. In the future, we intend to search for alloys of Mg that are more clinically applicable and for coatings that afford better control of the degradation of a scaffold.

## References

[1] Flierl MA, Smith WR, Mauffrey C, Irgit K, Williams AE, Ross E (2013). Outcomes and complication rates of different bone grafting modalities in long bone fracture nonunions: a retrospective cohort study in 182 patients. J Orthop Surg Res.

[2] Liang H, Li X, Shimer AL, Balian G, Shen FH (2014). A novel strategy of spine defect repair with a degradable bioactive scaffold preloaded with adipose-derived stromal cells. Spine.

[3] Brandoff JF, Silber JS, Vaccaro AR (2008). Contemporary alternatives to synthetic bone grafts for spine surgery. Am J Orthop.

[4] Feng W, Fu L, Liu J, Li D (2012). The expression and distribution of xenogeneic targeted antigens on porcine bone tissue. Transplant Proc.

[5] Clemens MW, Chang EI, Selber JC, Lewis VO, Oates SD, Chang DW (2012). Composite extremity and trunk reconstruction with vascularized fibula flap in postoncologic bone defects: a 10-year experience. Plast Reconstr Surg.

[6] Gil-Albarova J, Gil-Albarova R (2012). Donor site reconstruction in iliac crest tricortical bone graft: surgical technique. Injury.

[7] Chadha M, Arora SS, Singh AP, Gulati D, Singh AP (2010). Autogenous non-vascularized fibula for treatment of giant cell tumor of distal end radius. Arch Orthop Trauma Surg.

[8] Cobos JA, Lindsey RW, Gugala Z (2000). The cylindrical titanium mesh cage for treatment of a long bone segmental defect: description of a new technique and report of two cases. J Orthop Trauma.

[9] Segal U, Shani J (2010). Surgical management of large segmental femoral and radial bone defects in a dog: through use of a cylindrical titanium mesh cage and a cancellous bone graft. Vet Comp Orthop Traumatol.

[10] Ostermann PA, Haase N, Rübberdt A, Ekkernkamp A (2002). Management of a long segmental defect at the proximal meta-diaphyseal junction of the tibia using a cylindrical titanium mesh cage. J Orthop Trauma.

[11] Lindsey RW, Gugala Z, Milne E, Sun M, Gannon FH, Latta LL (2006). The efficacy of cylindrical titanium mesh cage for the reconstruction of a critical-size canine segmental femoral diaphyseal defect. J Orthop Res.

[12] Clements JR, Carpenter BB, Pourciau JK (2008). Treating segmental bone defects: a new technique. J Foot Ankle Surg.

[13] Wu L, Feyerabend F, Schilling AF, Willumeit-Römer R, Luthringer BJ (2015). Effects of extracellular magnesium extract on the proliferation and differentiation of human osteoblasts and osteoclasts in coculture. Acta Biomater.

[14] Witte F, Ulrich H, Palm C, Willbold E (2007). Biodegradable magnesium scaffolds: part II: peri-implant bone remodeling. J Biomed Mater Res A.

[15] Castellani C, Lindtner RA, Hausbrandt P, Tschegg E, Stanzl-Tschegg SE, Zanoni G (2011). Bone-implant interface strength and osseointegration: biodegradable magnesium alloy versus standard titanium control. Acta Biomater.

[16] Witte F, Kaese V, Haferkamp H, Switzer E, Meyer-Lindenberg A, Wirth CJ (2005). In vivo corrosion of four magnesium alloys and the associated bone response. Biomaterials.

[17] Guo JW, Sun SY, Wang YM, Zhou Y, Wei DQ, Jia DC (2015). Hydrothermal biomimetic modification of micro-arc oxidized magnesium alloy for enhanced corrosion resistance and deposition behaviors in SBF. Surf Coat Technol.

[18] Schmitz JP, Hollinger JO (1986). The critical size defect as an experimental model for craniomandibulofacial nonunions. Clin Orthop.

[19] Lane J, Sandhu H (1987). Current approaches to experimental bone grafting. Orthop Clin N Am.

[20] Chaya A, Yoshizawa S, Verdelis K, Myers N, Costello BJ, Chou DT (2015). In vivo study of magnesium plate and screw degradation and bone fracture healing. Acta Biomater.

[21] Zaky SH, Lee KW, Gao J, Jensen A, Close J, Wang Y (2014). Poly (glycerol sebacate) elastomer: a novel material for mechanically loaded bone regeneration. Tissue Eng Part A.

[22] Passos SP, Nychka JA, Major P, Linke B, Flores-Mir C (2015). In vitro fracture toughness of commercial Y-TZP ceramics: a systematic review. J Prosthodont.

[23] Guan F, Ma S, Shi X, Ma X, Chi L, Liang S (2014). Biocompatibility of nano-hydroxyapatite/Mg-Zn-Ca alloy composite scaffolds to human umbilical cord mesenchymal stem cells from Wharton’s jelly in vitro. Sci China Life Sci.

[24] Hofstetter J, Martinelli E, Pogatscher S, Schmutz P, Povoden- Karadeniz E, Weinberg AM (2015). nfluence of trace impurities on the in vitro and in vivo degradation of biodegradable Mg-5Zn-0.3Ca alloys. Acta Biomater.

[25] Qu Y, Kang M, Dong R, Liu J, Liu J, Zhao J (2015). Evaluation of a new Mg-Zn-Ca-Y alloy for biomedical application. J Mater Sci Mater Med.

[26] Park RS, Kim YK, Lee SJ, Jang YS, Park IS, Yun YH (2012). Corrosion behavior and cytotoxicity of Mg-35Zn-3Ca alloy for surface modified biodegradable implant material. J Biomed Mater Res B Appl Biomater.

[27] Ma WH, Liu YJ, Wang W, Zhang YZ (2015). Improved biological performance of magnesium by micro-arc oxidation. Braz J Med Biol Res.

[28] Li H, Pan H, Ning C, Tan G, Liao J, Ni G (2015). Magnesium with micro-arc oxidation coating and polymeric membrane: an in vitro study on microenvironment. J Mater Sci Mater Med.

[29] Xiong Y, Hu Q, Song R, Hu X (2017). LSP/MAO composite bio-coating on AZ80 magnesium alloy for biomedical application. Mater Sci Eng C Mater Biol Appl.

[30] Song G, Atrens A, Dargusch M (1998). Influence of microstructure on the corrosion of die cast AZ91D. Corros Sci.

[31] Pan Y, He S, Wang D, Huang D, Zheng T, Wang S (2015). In vitro degradation and electrochemical corrosion evaluations of microar oxidized pure Mg, Mg-Ca and Mg-Ca-Zn alloys for biomedical applications. Mater Sci Eng C Mater Biol Appl.

[32] Yoshizawa S, Brown A, Barchowsky A, Sfeir, Magnesium C (2014). Ion stimulation of bone marrow stromal cells enhances osteogenic activity, simulating the effect of magnesium alloy degradation. Acta Biomater.

[33] Guo Y, Ren L, Liu C, Yuan Y, Lin X, Tan L (2013). Effect of implantation of biodegradable magnesium alloy on: BMP-2 expression in bone of ovariectomized osteoporosis rats. Mater Sci Eng C Mater Biol Appl.

[34] Fazel Anvari-Yazdi A, Tahermanesh K, Hadavi SM, Talaei-Khozani T, Razmkhah M, Abed SM (2016). Cytotoxicity assessment of adipose-derived mesenchymal stem cells on synthesized biodegradable Mg-Zn-Ca alloys. Mater Sci Eng C Mater Biol Appl.

[35] Janning C, Willbold E, Vogt C, Nellesen J, Meyer-Lindenberg A, Windhagen H (2010). Magnesium hydroxide temporarily enhancing osteoblast activity and decreasing the osteoclast number in peri-implant bone remodelling. Acta Biomater.

[36] Chen S, Guan S, Li W, Wang H, Chen J, Wang Y (2012). In vivo degradation and bone response of a composite coating on Mg-Zn-Ca alloy prepared by microarc oxidation and electrochemical deposition. J Biomed Mater Res B Appl Biomater.

[37] Smith MR, Atkinson P, White D, Piersma T, Gutierrez G, Rossini G (2012). Design and assessment of a wrapped cylindrical Ca-P AZ31 Mg alloy for critical-size ulna defect repair. J Biomed Mater Res B Appl Biomater.

[38] Zou J, Shi Z, Xu H, Li X (2017). In vitro studies on the degradability, bioactivity, and cell differentiation of PRP/AZ31B Mg alloys composite scaffold. Biomed Res Int.

[39] Yu W, Zhao H, Ding Z, Zhang Z, Sun B, Shen J (2017). In vitro and in vivo evaluation of MgF2 coated AZ31 magnesium alloy porous scaffolds for bone regeneration. Colloids Surf B Biointerfaces.

[40] Gu XN, Xie XH, Li N, Zheng YF, Qin L (2012). In vitro and in vivo studies on a Mg-Sr binary alloy system developed as a newkind of biodegradable metal. Acta Biomater.

